# Risk of serotonin syndrome in acutely ill patients receiving linezolid and opioids concomitantly: a retrospective cohort study

**DOI:** 10.1016/j.ijregi.2022.09.008

**Published:** 2022-09-24

**Authors:** Hassan Mitwally, Mohamed Omar Saad, Dania Alkhiyami, Amr Mohamed Fahmi, Sara Mahmoud, Eman Al Hmoud, Rasha El Enany, Hassan Younis, Shaban Mohammed, Palli Abdul Rouf, Binny Thomas, Moza Al Hail

**Affiliations:** 1 Pharmacy Department, Al-Wakra Hospital, Hamad Medical Corporation, Qatar; 2 Pharmacy Department, Heart Hospital, Hamad Medical Corporation, Qatar; 3 Pharmacy Department, Hamad Medical Corporation, Qatar

**Keywords:** linezolid, opioid, serotonin syndrome, monoamine oxidase inhibitor, drug interaction

## Abstract

**Introduction:**

Linezolid is an oxazolidinone antibiotic with a reversible, non-selective, monoamine oxidase inhibitory effect. Combining linezolid with serotonergic agents may increase serotonin syndrome (SS) risk.

Linezolid is recommended in patients with suspected or confirmed resistant Gram-positive bacterial infections, especially if vancomycin cannot be used. However, it is unclear whether co-administration of linezolid with opioids increases the risk of serotonin syndrome.

**Research objective:**

To establish whether combining linezolid with opioids will increase the incidence of SS in acutely ill patients.

**Methods:**

This was a retrospective observational study. All adult patients who were admitted and received linezolid between March and September 2020 were included in the study. The primary outcome was the prevalence of SS, as defined by Hunter's criteria.

**Results:**

The study included 106 patients, most whom were males (91.5%). More than half of the cohort (56.6%) received a concomitant opioid agent. Morphine and fentanyl were the most prescribed opioids (37.7% and 34%, respectively). Among patients who received opioids, only one patient (1.6%) had spontaneous clonus. However, this patient developed spontaneous clonus post cardiac arrest, which made an association with the linezolid–opioids combination less likely.

**Conclusion:**

In this study, the incidence of SS was low in acutely ill patients who received concomitant linezolid and opioids. However, larger prospective studies are required to confirm this finding.

## Highlights

• The incidence of serotonin syndrome is low in patients receiving linezolid and opioids.
• Administration of linezolid and opioids should not be avoided if indicated.
• Other patients’ conditions may interfere with the diagnosis of serotonin syndrome.

## Introduction

Serotonin syndrome is a life-threatening adverse drug reaction that is characterized by a triad of neuroexcitatory features, due to excess serotonergic activity at central receptors (Boyer and Shannon, 2005). These characteristic symptoms comprise neuromuscular hyperactivity, autonomic hyperactivity, and altered mental status (Gillman, 2005). Clinical features can range from mild to life-threatening, and the onset is usually rapid, within hours of drug combinations, although there have been reported cases with delayed reactions (Haddad et al., 2013). Serotonergic drug classes include selective serotonin reuptake inhibitors (SSRIs), tricyclic antidepressants, MAOIs, opioids, and triptans (Boyer and Shannon, 2005). The most common drug combination that can cause this syndrome is a monoamine oxidase inhibitor (MAOI) drug in conjunction with any serotonin reuptake inhibitor (SRI) (Gillman, 2005). Thus, the use of opioids in combination with MAOIs, or within a 2-week period following the discontinuation of an irreversible MAOI, should be avoided when possible (Aronson, 2015).

Linezolid is an oxazolidinone antibiotic with a weak, reversible, non-selective MAOI (British National Formulary). It is predicted that combinations of linezolid with various SRIs, such as opioids, might precipitate serotonin syndrome (Moellering, 2003). There is limited evidence on the interaction between linezolid and opioids, with only a few cases reported; thus, it is difficult to draw any definite conclusions (Das et al., 2008; Lawrence et al., 2006; Moellering, 2003). Moreover, there is conflicting clinical evidence in the literature about the degree and risk of interaction between linezolid and opioids such as fentanyl and morphine, with some reviews suggesting that fentanyl is safe when co-administered with MAOIs (Huang and Gortney, 2006; Lum and Stahl, 2012).

Linezolid is a therapeutic option in managing bacterial infections with multidrug-resistant Gram-positive organisms, such as methicillin-resistant *Staphylococcus aureus* (MRSA) (Kalil et al., 2013). Linezolid is the drug of choice in treating patients with vancomycin-resistant enterococcus infections or in patients with MRSA infections and renal insufficiency, where administration of nephrotoxic medication such as vancomycin may worsen the kidney injury, especially if co-administered with another nephrotoxic drug (Hirai et al., 2019).

However, linezolid and opioids are essential medications, especially for acutely or critically ill patients, and the concomitant administration of both agents may be of significant clinical importance. To our knowledge, there has been no study done to evaluate the risk of interaction between linezolid and opioids in acutely ill patients.

## Objective

The purpose of this study was to evaluate the incidence of serotonin syndrome among acutely ill patients who received concomitant opioids and linezolid.

## Patients and methods

### Study design and population

A retrospective cohort study was carried out, which included adult patients (> 18 years of age) admitted to one of the Hamad Medical Corporation (HMC) COVID-19 facilities, who were hospitalized between March 2020 and September 2020, and who received concomitant linezolid and opioids. Patients were excluded if they received linezolid for less than one day. Patients were followed up for 2 weeks after the start of linezolid administration.

### Data collection

The following information was collected from the electronic medical records of eligible patients: demographics, comorbid diseases, spontaneous clonus, temperature > 38°C, tremor and hyperreflexia, intubation status, intensive care unit (ICU) admission and discharge dates, mortality, and other medications that can cause serotonin syndrome, according to the Lexicomp drug information handbook. All opioids approved in our hospitals were included: morphine, fentanyl, remifentanil, and tramadol.

### Assessment of outcomes

The primary outcome was the incidence of serotonin syndrome (SS) according to the patients’ clinical findings. Serotonin syndrome was defined according to Hunter's criteria. These state that for a patient to be diagnosed with SS, the patient should be receiving a serotonergic agent and develops one of the following: spontaneous clonus, inducible clonus *plus* agitation or diaphoresis, ocular clonus *plus* agitation or diaphoresis, tremor *plus* hyperreflexia, or hypertonia *plus* temperature above 38°C *plus* ocular clonus or inducible clonus.

### Statistical analysis

The primary outcome of serotonin syndrome incidence was presented as frequency and percentage. The patients’ characteristics were presented as frequencies with percentages for categorical characteristics and means with standard deviations for continuous characteristics. All statistical analyzes were performed using the Statistical Package for Social Sciences (SPSS) program version 25 (IBM Corp., Armonk, NY).

## Results

In total, 106 acutely ill patients were included, with a mean age (± SD) of 53 ± 16.2 years. Ninety-seven patients (91.5%) were males. Diabetes, hypertension, and chronic kidney disease were the most common comorbidities (51.9%, 44.3%, and 19.8%, respectively). Table 1 presents the demographic characteristics of the study population. Ninety-four patients (88.67%) were under ICU care, with more than half of these (53.8%) requiring invasive mechanical ventilation. The 30-day mortality was 36.8%, which reflects how critically ill these patients were. All patients who received concomitant linezolid and opioids were in ICU.

**Table 1:** Patient demographics and medical data

Characteristic	*N* = 106
Age, mean ± SD	53 ± 16.2
Male sex, *n* (%)	97 (91.5)
Diabetes mellitus, *n* (%)	55 (51.9)
Hypertension, *n* (%)	47 (44.3)
Chronic kidney disease, *n* (%)	21 (19.8)
Chronic liver disease, *n* (%)	5 (4.7)
Chronic respiratory disease, *n* (%)	19 (17.9)
Malignancy, *n* (%)	9 (8.5)
ICU admission, *n* (%)	94 (88.67)
Invasive mechanical ventilation, *n* (%)	57 (53.8)
Mortality, *n* (%)	39 (36.8)
Route of linezolid administration, *n* (%)	
Oral/NG	35 (33)
Intravenous	69 (65.1)
Oral/NG/IV	2 (1.9)
Number of serotonergic medications received during linezolid therapy, *n* (%)	
0	38 (35.8)
1	43 (40.6)
2	17 (16)
3	8 (7.5)
Concomitant medications, *n* (%)	
Any opioids	60 (56.6)
Morphine	40 (37.7)
Fentanyl	36 (34)
Dextromethorphan	11 (10.4)
Remifentanil	11 (10.4)
Ondansetron	2 (1.9)
Amitriptyline	1 (0.9)

SD: standard deviation, ICU: intensive care unit, NG: nasogastric, IV: intravenous

Linezolid was administered intravenously for 69 patients (65.1%). Sixty-eight patients (64%) were prescribed concomitant linezolid and serotonergic agents. Sixty patients (56.6%) received a combination of linezolid and opioids. Morphine and fentanyl were the two commonly prescribed opioids (37.7% and 34% of the total serotonergic drugs, respectively). The other serotonergic drugs included ondansetron (1.9%) and amitriptyline (0.9%) (Table 1).

Among patients who received opioids, only one patient met the SS criteria (1.6%), manifested as spontaneous clonus. However, this patient developed spontaneous clonus post cardiac arrest, which made the association between SS and the linezolid–opioids combination less likely (Table 2).

**Table 2:** Hunter's criteria for patients receiving concomitant linezolid and opioids

Criteria	*n* (%)
Spontaneous clonus	1 (1.66)
Inducible clonus and agitation or diaphoresis	0 (0)
Ocular clonus and agitation or diaphoresis	0 (0)
Tremor and hyperreflexia	0 (0)
Temperature of 38°C and ocular clonus or inducible clonus	0 (0)

## Discussion

The purpose of this study was to evaluate the incidence of serotonin syndrome (SS) among acutely ill patients who received concomitant linezolid and opioids. The incidence of SS was very low: among 60 patients who received concomitant linezolid and opioids, only one patient met Hunter's criteria. Although they had received concomitant linezolid and fentanyl, the patient presented spontaneous myoclonus after cardiac arrest, which made an association between SS and linezolid–opioid drug interaction unlikely.

Severe, life-threatening SS may be associated with high-grade fever (> 41°C), seizures, coma, arrhythmia, rhabdomyolysis, metabolic acidosis, respiratory failure, and death (Boyer and Shannon, 2005; Volpi-Abadie et al., 2013). Although mild, undetected forms of SS could not be ruled out in this study, severe SS secondary to an opioids–linezolid combination was not observed in this patient cohort. Hunter's criteria were used to evaluate the incidence of SS; these became the gold standard for diagnosing SS because they were more sensitive than the Sternbach criteria (84% versus 75%, respectively) (Dunkley et al., 2003).

SS due to a combination of linezolid and opioids has been observed and reported in a few cases (Mastroianni et al., 2017; Samartzis et al., 2013). One case report led to a high suspicion of SS due to the co-administration of linezolid and methadone (Mastroianni et al., 2017). Another case report showed that a combination of linezolid, fentanyl, and amitriptyline resulted in SS. However, in this case, there was administration of a third serotonergic agent, which may have augmented the incidence of SS (Samartzis et al., 2013).

Butterfield et al., 2012 conducted an analysis of phase III and IV randomized clinical trials data, evaluating serotonin toxicity with concomitant use of either linezolid or comparators and serotonergic agents (Butterfield et al., 2012). Three patients (0.14%) fulfilled Hunter's criteria in the linezolid group. Although 22% of patients in each arm received analgesics, including opioids and non-opioid agents, the authors did not specify whether these three patients received concomitant opioids or not. Additionally, most patients had comorbidities, which may have contributed to the reported adverse events. A recently published cross-sectional study analyzed the risk of SS in 106 patients who received 3 days or more of linezolid and either methadone or buprenorphine (Traver et al., 2022). In line with our study, the incidence of SS was low; no cases met the criteria for definite SS, and two cases met the criteria for possible SS; neither of these cases met Hunter's criteria for SS. However, the study included only two opioids, with 85% of patients receiving linezolid with methadone, which may weaken the generalizability of these data.

The worry of SS induced by opioids–linezolid drug–drug interaction may be enough reason to deprive acutely ill patients of a safe and effective therapeutic option — linezolid. On the other hand, opioids are commonly required for sedation and analgesia, especially for intubated patients or those undergoing invasive procedures. Evaluating the incidence and severity of this drug–drug interaction is crucial for a better understanding of linezolid safety in acutely ill patients receiving opioids.

To the best of our knowledge, this study was the first designed to evaluate the incidence of SS among acutely ill patients who received concomitant linezolid and different opioid analgesics. This study had some limitations. One of these was the observational study design, since all required data were collected retrospectively from the patients’ electronic files, and some patients may have developed SS signs or symptoms that were missed or not documented by the physician or the bedside nurse. Another limitation was the sample size, since the study included 106 patients, with only 60 receiving concomitant linezolid and opioids. The limited number of patients and the fact that this was a single-center study limit the generalizability of our findings. Additionally, this study was conducted in COVID-19 facilities during the COVID-19 pandemic. However, we believe that this should not restrict the generalizability of the results to non-COVID-19 acutely ill patients.

## Conclusion

According to our results, the incidence of SS among acutely ill patients who received concomitant opioids and linezolid was very low, and the use of this combination is probably safe. Further prospective studies with a larger sample size are needed to confirm these findings.

## Conflicts of interest

The authors declare that they have no conflicts of interest to disclose.
